# Tai Chi and Workplace Wellness for Health Care Workers: A Systematic Review

**DOI:** 10.3390/ijerph17010343

**Published:** 2020-01-03

**Authors:** Rosario Andrea Cocchiara, Barbara Dorelli, Shima Gholamalishahi, William Longo, Emiliano Musumeci, Alice Mannocci, Giuseppe La Torre

**Affiliations:** Department of Public Health and Infectious Diseases, Sapienza University, Piazzale Aldo Moro, 5-00185 Rome, Italy; barbara.dorelli@uniroma1.it (B.D.); shima.gholamalishahi@uniroma1.it (S.G.); longo.1708779@studenti.uniroma1.it (W.L.); musumeci.632908@studenti.uniroma1.it (E.M.); alice.mannocci@uniroma1.it (A.M.); giuseppe.latorre@uniroma1.it (G.L.T.)

**Keywords:** tai chi, workplace wellness, workplace wellbeing, nursing, health professional, stress

## Abstract

Several studies show the positive effects of new non-medical therapies known as complementary and alternative medicines (CAMs). In this context, the discipline of tai chi is obtaining a wider consensus because of its many beneficial effects both on the human body and mind. The aim of this study was to perform a systematic review of the scientific literature concerning the relationship between tai chi practice and wellness of health care workers (HCW) in their professional setting. The research was performed in September 2019, investigating the databases Cinahl, Scopus, Web of Science, and PubMed. Full-text articles, written in English language and published after 1995, were taken into account. No restrictions regarding the study design were applied. A quality assessment was developed using AMSTAR, Jadad, Newcastle–Ottawa Scale, INSA, and CASE REPORT scale. Six papers were finally included: Three clinical trials, one observational study, one systematic review, and one case report. The methodological quality of the included studies was judged as medium level. In conclusion, this systematic review suggests the potential impact of interventions such as tai chi as tools for reducing work-related stress among healthcare professionals. Further research will be needed in order to gain robust evidence of its efficacy.

## 1. Introduction 

Stress identifies the body’s reaction to any change that requires a physical, mental, or emotional response. The World Health Organization (WHO) states that mental health problems such as stress will likely become the second most common disability by 2020 [1]. Mind–body exercises are increasingly recognized as beneficial for physical, emotional, and mental health [2]. There are many factors that can contribute to make the work of healthcare providers stressful; dealing with death, lack of resources, high workload, and difficult and unpleasant collaboration in teamwork might negatively influence health and work performance of healthcare staff [3]. In particular, nurses have high levels of stress that affect the duration of care, personal wellness, and efficiency [4]. Out of 2500 nurses, approximately 75% of them reported working with pain, and nearly 20% also showed depressive symptoms [5]. The implemented strategies to improve the health and wellbeing of nursing staff are important, since their work has a direct effect on patients [5]. Disturbance in mental health has become the main health concern in society, and tai chi seems a valid tool to solve it [1]. Tai chi is an ancient Chinese martial art that has attracted growing interest. It is an aerobic exercise of mild to moderate intensity, and its practice involves an interaction of physical movement, meditation, and deep breathing [6]. As it comprises mental concentration, physical balance, muscle relaxation, and relaxed breathing, tai chi shows a great potential for becoming widely integrated into prevention and rehabilitation interventions for a great number of medical and psychological conditions [6]. The scientific literature highlights that this discipline improves the quality of life by reducing stress, intervening simultaneously on the mind and body [2]. In fact, the positive effects of tai chi have been widely demonstrated on many pathologies, and the interest in its benefits has exponentially grown along with the number of individuals practicing it [7]. During the exercise, an increase in the synthesis of serotonin and dopamine has been demonstrated [6]; moreover, improvements in the function of the cardiovascular system, type II diabetes, and musculoskeletal disabilities have been recorded [8]. Due to these effects, its application would be appropriate for healthcare professionals to increase their mental wellbeing, reduce stress and enhance concentration and relaxation; furthermore, it would impact on their physical functioning, improving muscle tension and joint mobility. However, little is known concerning the efficacy of tai chi in reducing work-related stress among health professionals, and results have not been conclusive [9]. The aim of this study was to carry out a systematic review of the literature concerning the use of tai chi in reducing stress in health care workers.

## 2. Materials and Methods

### 2.1. Search Strategy

**Figure 1:**
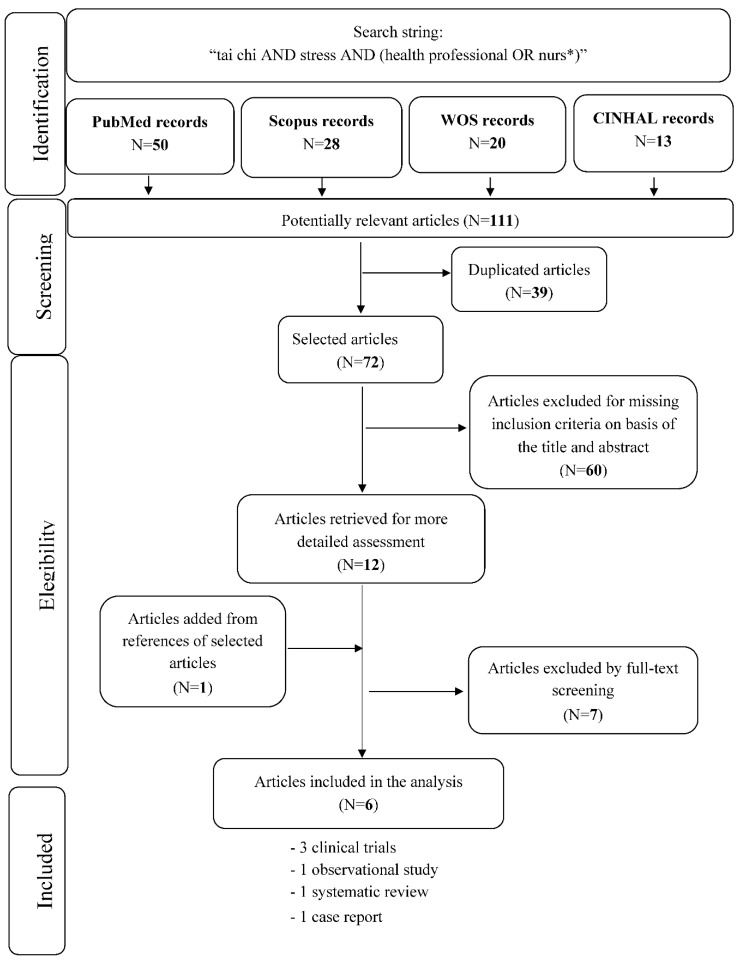
Flow-chart of search strategy.

This systematic review was performed on the basis of the Preferred Reporting Items for Systematic Reviews and Meta-Analysis (PRISMA) statement [10]. In order to collect data from the medical, nursing, and scientific literature, the following databases were searched on March 2019: PubMed, Scopus (Elsevier), Cinahl, and Web of Science. Articles were retrieved using the string: “tai chi AND stress AND (health professional OR nurs*)”. Backward citation search was performed in order to ensure a wide access to previous scientific evidence that might have not been detected by our search string. Figure 1 illustrates the flow-chart relating to the literature search strategy.

### 2.2. Study Selection

Studies were recruited if they focused on the positive effects of tai chi on the psychophysical wellbeing of the healthcare workers. Studies were included if they were published in English after 1995. No restrictions to study design were applied. The objective of the study was addressed by referring to the PICOS (Population, Intervention, Comparison, Outcome, Study design) questions: The population was identified in health professionals; the intervention in object was tai chi; the comparator was alternative techniques (such as yoga or traditional care); the outcomes were reduced work-related stress, better physical and psychological function (e.g., anxiety, depression), improvement in attention and/or productivity; the study design was pilot studies, observational studies, randomized clinical trials (RCTs), narrative, and systematic reviews.

Studies were assessed for eligibility through a multi-step approach: Screening by title, abstract, and full text. Study selection was performed independently by two different authors and then compared in order to reduce risk of assessment bias. Discrepancies in the evaluation were solved by consensus with a third author.

### 2.3. Data Extraction

Included studies underwent data extraction collecting the following information: First author, country, study design, year of publication, number of participants or number of included studies, outcome. In order to reduce bias from authors, data extraction was performed by two researchers independently, and discrepancies process were solved by consensus with a third researcher.

### 2.4. Quality Assessment 

The methodological quality of all studies included in the systematic review was assessed. Quality assessment was performed according to the Newcastle–Ottawa Scale (NOS) on cohort or cross-sectional studies [11], the Jadad scale for randomized clinical trial [12], AMSTAR for systematic reviews [13], and CASE REPORT scale for case study [14].

## 3. Results

### 3.1. The Identification of Relevant Studies 

A total of 111 references were retrieved from the database investigation: 50 in PubMed, 28 in Scopus, 20 in Web of Science, and 13 in CINHAL. After the exclusion of duplicates (39 articles) the remaining 72 references underwent a title and abstract analysis, after which 60 were excluded. After full-text screening, five articles were included in the systematic review: Two clinical trials, one observational study, one systematic review, and one case report. One further clinical trial was identified from the analysis of the bibliographies of selected studies. Table 1 summarizes the characteristics of the included studies.

The study of Kemper and colleagues shows the results of an online survey administered to 342 North American nurses to evaluate their interest in meditation, prayer, and mind–body practices, such as yoga, tai chi, qigong, Zen, acupuncture, etc., in reducing stress. The e-survey included: Demographic characteristics, health conditions and stress levels, experiences with mind–body practices, expected health benefits, training preferences, and will to participate in future randomized controlled trials. The authors found a decline of the quality of life with evident dissatisfaction; most of the respondents (73%) reported one or more health conditions, more specifically 49% of the nurses reported anxiety disorders, 41% back pain, 34% gastrointestinal disorders, and 33% depression. The number of burnout cases was also increasing. These disorders were perceived as an high level of stress, since the assigned value in a scale from 0 to 5 was 4 (0 = none, 5 = extreme stress). Nearly all (99%) reported already using one or more mind–body practices to reduce stress and, among these, yoga/tai chi/qigong practices were the 34% [16]. The identified clinical trials are in line with these statistics and describe the effectiveness of these interventions. A pilot study was carried out by researchers in nursing at the University of Vermont [4] to assess the feasibility of a tai chi workplace wellness program as a cost effective way of improving physical and mental health, reducing work related stress, and improving work productivity among nurses over 49 years of age. Participants were divided in two groups: The tai chi group (*n* = 6) was asked to attend tai chi classes once a week offered at their worksite and to practice on their own for 10 minutes each day at least 4 days per week for 15 weeks. Controls (*n* = 5) received no intervention. After 15 weeks, the tai chi group showed an increase of 3% in productivity in the workplace and also an improvement in musculoskeletal pain and a greater reduction in work-related stress (−20% in NSS—Nursing Stress Scale) than the control group (−8.5%, *p* = 0.89). The tai chi group also showed a larger reduction in general stress (−23% in PSS—Perceived Stress Scale) than the control group (−17.5%, *p* = 0.42). Similarly, Steinberg and colleagues investigated the effect of a very short intervention of an eight-week tai chi program on 15 nurses. They found a significant improvement in sleep (*t* = −4.01, *p* = 0.007); stress reduction; and nurse desire to be competent, successful, and compassionate in their work [5]. 

Lastly, in 2018, Marshall et al. conducted a trial that consisted of a 12-week intervention of tai chi delivered to 12 healthcare professionals within the same healthcare center in order to encourage their participation and to minimize dropouts. In this study, wellbeing of participants was measured before and after the intervention using the WEMWBS scale. This research also demonstrated a significant increase of the general mental wellbeing of subjects, with a gain of mean score of 4.13 (*p* < 0.005) [17]. Moreover, the systematic review of Budhrani et al. shows evidence that, after a 15-week tai chi program on 11 nurses suffering from work-related musculoskeletal disorders, significant improvements in physical and mental health, higher reduction in work and general stress, and improvement in trunk flexibility were seen, compared to the control group [9].

However, although the reported studies refer to the nursing population, results of effectiveness are also confirmed in other professionals. For example, Benor in 1995 considered the Louisville program for medical students and showed that nutrition, exercise, and relaxation (muscle relaxation, music, art, song, tai chi and other methods) could play an important role for health maintenance and disease prevention in medical students [15].

### 3.2. Quality Assessment 

Clinical Trials were of poor quality, achieving zero, one, and three points on the Jadad scale out of five. The observational study and the case report were of average quality, with scores of 5/9 and 7/10, respectively. Low quality was observed in the systematic review (3/11). According to the quality assessment tools, the overall methodological quality is poor–medium level. The application of tai chi to improve health professionals’ wellbeing is still limited, and the absence of a standardized intervention (for example, in terms of length and structure of a tai chi session) impacts on the on the scarce methodology of the studies and reduces the robustness of the retrieved evidence.

## 4. Discussion

The literature produced to date on this topic is limited. After extensive research on major databases such as Scopus, PubMed, Cinahl, and Web of Science, we were able to find only six useful articles for this systematic review. Following the use of quality assessment tools such as AMSTAR, Jadad, Newcastle–Ottawa Scale, and CASE REPORT, we can say that the methodological quality of the published literature is of a poor level. 

Healthcare professions are among the most vulnerable for stress, and this is related to the characteristics of the work, with lack of adequate training, poor social recognition, excessive workloads, and bad organizational strategies [4]. Acute and prolonged stress, if untreated, leads to chronic symptoms that may not react to traditional therapies. For this reason, more and more often, individuals are pushed to undertake alternative non-medical pathways. The use of complementary and alternative medicines (CAMs) represents an useful tool to reduce stress-related chronic pain; among the most used CAMs there are food supplements (39.1%), chiropractic (23.4%), manual massages (23.1%), meditation/yoga/tai chi (20.5%), homeopathic remedies (12.8%), and acupuncture (4.7%) [18,19,20].

Since 1980, a low level of selfcare has been observed among health professionals, although the exposure of this work category to high risk of mental and physical deterioration has been recognized, so resulting in risky behaviors (alcohol or substances abuse, or suicide). Tai chi and other support techniques could represent valid tools to obtain stress reduction [15].

From the collected evidence, the positive effects of tai chi on many pathologies have been widely demonstrated, so a great scientific interest in its benefits has exponentially grown; moreover, the number of people who use it to improve their physical and mental health is increasing. A study published in 2018 has shown how tai chi has immediate positive effects on perceived attention and stress. This study compared a control group (*n* = 20) and an intervention group (*n* = 20) involved in a 10 minute-per-day tai chi program. Electroencephalogram analysis to assess attention and stress levels was performed for both groups at the beginning, during, and at the end of the study. From the collected data it was noticed that for the tai chi group, the levels of attention increased during the exercises and decreased immediately after the end. The levels of perceived stress after the exercises were also significantly lower than those at the beginning while among the control group all parameters remained unchanged [21]. There are many conditions in which its application could be beneficial: Back pain (41.3%), joint pain or arthritis (37.7%), headache (32.8%), insomnia (27.8%), weight loss (20.9%), hypertension (19.6%), depression (11.0%), osteoporosis (7.3%), cardiac disorders (4.6%), uterine fibroids (3.9%), during pregnancy (49.1%) [20]. Its positive effect has also been demonstrated in HIV-infected patients, women with early-stage breast cancer, in cardiovascular disease, osteoarthritis, type II diabetes, musculoskeletal conditions, depression, improved sleep and stress [22,23,24]; it also appears useful for improving cognitive functions and reducing pain [22]. 

Chan and colleagues compared tai chi exercise with brisk walking in its efficacy for reducing cardiovascular disease risk factors among adults with hypertension. This trial confirms the efficacy of tai chi since, after nine months, a significant reduction in blood pressure (systolic −12.46 mmHg; diastolic −3.20 mmHg), fasting blood sugar (−1.27 mmol/L), glycated hemoglobin (−0.56%), and perceived stress (−2.32 score) were observed. Furthermore, participants showed improved perceived mental health (+3.54 score) and exercise self-efficacy (+12.83 score) [25].

Tai chi’s clinical validity has been demonstrated in more than one field, and it is also easily reproducible as it is a low-risk economic activity with multiple physical and psychological advantages, but a deepening of the studies is now necessary [26]. This ancient martial art is an activity that involves both the body and the mind. The physical exercises of body balance are combined with meditation courses focused on breathing [24,27]; this synergy favorably predisposes to concentration by temporarily diverting attention from the stress of daily life.

The weaknesses of this analysis are undeniably related to the low numerical consistency of studies present in the literature and, at the same time, to the heterogeneity of the interventions that result in a difficulty to make any comparison. The studies included in this review are relatively recent but of poor quality.

Conversely, the strengths of this study are represented by the revolution that this type of approach brings for the management of healthcare workers, as the costs for implementing them are low in comparison to the potential health benefits.

## 5. Conclusions

According to the published literature, tai chi represents a valid tool to improve many pathological conditions, and it could reduce work-related stress as well. Although present data highlight the full potential and possible benefits derived from these techniques, in order to warrant a widespread diffusion, it would be necessary to further deepen scientific evidence by designing and implementing research studies that could confirm tai chi’s beneficial effects for the wellbeing of healthcare professionals.

## Figures and Tables

**Table 1:** Characteristics of the included studies.

Authors	Country	Study Design	Number of Participants or Number of Paper Included	Year of Publication	Results	Quality Assessment
Benor D.J [15]	UK	Case report	Not applicable	1995	This study identified nutrition, exercise, relaxations (including music, art, and tai chi) as important methods for health maintenance and disease prevention among medical student.	7/10 *****
Kemper K et al. [16]	USA	Observational study	342	2011	Mind–body practices including meditation, prayer, yoga, tai chi, and qigong to reduce stress and anxiety in nurses.	5/9 *
Marshall D et al. [17]	Ireland	Clinical Trial	12	2018	A 12-session intervention of tai chi was administered to a group of healthcare workers. A significant increase in these individuals’ wellbeing was measured comparing pre- and post-intervention measurements.	0/5 ***
Palumbo M et al. [4]	USA	Clinical Trial	14	2012	The tai chi group registered significantly fewer absence rates and 3% increase in productivity. No significant differences in physical or mental health scores (SF-12) were detected.	1/5 ***
Steinberg B et al. [5]	USA	Clinical Trial	15	2017	A very short intervention resulted in significant improvements in sleep quality, stress levels, and nursing staff’s motivation in their work.	3/5 ***
Budhrani-Shani P et al. [9]	USA	Systematic review	83	2016	After a 15-week tai chi program, significant improvements in physical and mental health were recorded, along with a significative reduction in stress levels were highlighted and an improvement in trunk flexibility.	3/11 **

* Newcastle–Ottawa Scale, ** AMSTAR scale, *** Jadad scale, ***** CASE REPORT.

## References

[1] Zheng S., Kim C., Lal S., Meier P., Sibbritt D., Zaslawski C. (2018). The Effects of Twelve Weeks of Tai Chi Practice on Anxiety in Stressed but Healthy People Compared to Exercise and Wait-List Groups–A Randomized Controlled Trial. J. Clin. Psychol..

[2] Love M.F., Sharrief A., Chaoul A., Savitz S., Beauchamp J.E.S. (2019). Mind-Body Interventions, Psychological Stressors, and Quality of Life in Stroke Survivors. Stroke.

[3] Gollwitzer P.M., Mayer R., Frick C., Oettingen G. Promoting the Self-Regulation of Stress in Health Care Providers: An Internet-Based Intervention. https://www.ncbi.nlm.nih.gov/pmc/articles/PMC6013563/.

[4] Palumbo M.V., Wu G., Shaner-McRae H., Rambur B., McIntosh B. (2012). Tai Chi for older nurses: A workplace wellness pilot study. Appl. Nurs. Res..

[5] Steinberg B., Bartimole L., Habash D., Fristad M.A. (2017). Tai Chi for Workplace Wellness: Pilot Feasibility Study. Explore.

[6] Zou L., Sasaki J.E., Wei G.X., Huang T., Yeung A.S., Barbosa Neto O., Chen K.W., Sai-chuen Hui S. (2018). Effects of Mind-Body Exercises (Tai Chi/Yoga) on Heart Rate Variability Parameters and Perceived Stress: A Systematic Review with Meta-Analysis of Randomized Controlled Trials. J. Clin. Med..

[7] Farhang M., Miranda-Castillo C., Rubio M., Furtado G. (2019). Impact of mind-body interventions in older adults with mild cognitive impairment: A systematic review. Int. Psychogeriatr..

[8] Fetherston C.M., Wei L. (2011). The benefits of tai chi as a self management strategy to improve health in people with chronic conditions. J. Nurs. Healthc. Chronic Illn..

[9] Budhrani-Shani P., Berry D.L., Arcari P., Langevin H., Wayne P.M. (2016). Mind-Body Exercises for Nurses with Chronic Low Back Pain: An Evidence-Based Review. Nurs. Res. Pract..

[10] Liberati A., Altman D.G., Tetzlaff J., Mulrow C., Gøtzsche P.C., Ioannidis J.P., Clarke M., Devereaux P.J., Kleijnen J., Moher D. (2009). The PRISMA statement for reporting systematic reviews and meta-analyses of studies that evaluate health care interventions: Explanation and elaboration. J. Clin. Epidemiol..

[11] Wells G.A., Shea B., O’Connell D., Peterson J., Welch V., Losos M., Tugwell P. (2015). The Newcastle-Ottawa Scale (NOS) for Assessing the Quality if Nonrandomized Studies in Meta-Analyses. http://www.ohri.ca/programs/clinical_epidemiology/oxford.htm.

[12] Jadad A.R., Moore R., Carroll D., Jenkinson C., Reynolds D.M., Gavaghan D.J., McQuay H.J. (1996). Assessing the quality of reports of randomized clinical trials: Is blinding necessary?. Control. Clin. Trials.

[13] Shea B.J., Grimshaw J.M., A Wells G., Boers M., Andersson N., Hamel C., Porter A.C., Tugwell P., Moher D., Bouter L.M. (2007). Development of AMSTAR: A measurement tool to assess the methodological quality of systematic reviews. BMC Med. Res. Methodol..

[14] Pierson D.J. (2009). How to read a case report (or teaching case of the month). Respir. Care.

[15] Benor D.J. (1995). The Louisville programme for medical student health awareness. Complement. Ther. Med..

[16] Kemper K., Bulla S., Krueger D., Ott M.J., McCool J.A., Gardiner P. (2011). Nurses’ experiences, expectations, and preferences for mind-body practices to reduce stress. BMC Complement. Altern. Med..

[17] Marshall D., Donohue G., Morrissey J., Power B. (2018). Evaluation of a Tai Chi Intervention to Promote Well-Being in Healthcare Staff: A Pilot Study. Religions.

[18] Ives J.C., Sosnoff J. (2000). Beyond the mind-body exercise hype. Physician Sportsmed..

[19] Chismark A., Asher G., Stein M., Tavoc T., Curran A. (2011). Use of complementary and alternative medicine for work-related pain correlates with career satisfaction among dental hygienists. J. Dent. Hyg..

[20] Wade C., Chao M., Kronenberg F., Cushman L., Kalmuss D. (2008). Medical Pluralism among American Women: Results of a National Survey. J. Womens Health (Larchmt).

[21] Cheung T.C.Y., Liu K.P.Y., Wong J.Y.H., Bae Y.H., Hui S.S., Tsang W.W.N., Cheng Y.T.Y., Fong S.S.M. (2018). Acute Effects of Tai Chi Training on Cognitive and Cardiovascular Responses in Late Middle-Aged Adults: A Pilot Study. Evid. Based Complement. Altern. Med..

[22] Tsai P.F., Kitch S., Chang J.Y., James G.A., Dubbert P., Roca J.V., Powers C.H. (2018). Tai Chi for Posttraumatic Stress Disorder and Chronic Musculoskeletal Pain: A Pilot Study. J. Holist. Nurs..

[23] Lorenc A., Feder G., MacPherson H., Little P., Mercer S.W., Sharp D. (2018). Scoping review of systematic reviews of complementary medicine for musculoskeletal and mental health conditions. BMJ Open.

[24] Robins J.L., Elswick R.K., McCain N.L. (2012). The story of the evolution of a unique tai chi form: Origins, philosophy, and research. J. Holist. Nurs..

[25] Chan A.W.K., Chair S.Y., Lee D.T.F., Leung D.Y.P., Sit J.W.H., Cheng H.Y., Taylor-Piliae R.E. (2018). Tai Chi exercise is more effective than brisk walking in reducing cardiovascular disease risk factors among adults with hypertension: A randomised controlled trial. Int. J. Nurs. Stud..

[26] Wang W.C., Zhang A.L., Rasmussen B., Lin L.W., Dunning T., Kang S.W., Park B.J., Lo S.K. (2009). The effect of Tai Chi on psychosocial well-being: A systematic review of randomized controlled trials. J. Acupunct. Meridian Stud..

[27] Holmberg M., Perdue A. (2013). How Tai Chi Is Your Nursing Staff?. Ky. Nurse.

